# Predictors of Outcome after Direct Aspiration of Basilar Artery Occlusion

**DOI:** 10.3390/jcm13061576

**Published:** 2024-03-09

**Authors:** Miroslav Mako, Georgi Krastev, Vladimír Nosáľ, Jozef Haring, Denisa Jakubcová, Martin Daniš, Andrej Klepanec, Ján Haršány, Štefan Sivák, Egon Kurča

**Affiliations:** 1 Department of Neurology, Faculty Hospital Trnava, 91701 Trnava, Slovakia; miroslav.mako@fntt.sk (M.M.); georgi.krastev@fntt.sk (G.K.); haring.jozef@fntt.sk (J.H.); danis.martin@fntt.sk (M.D.); 2 Slovak Medical University, Limbová 12, 83303 Bratislava, Slovakia; 3 Jessenius Faculty of Medicine in Martin, Comenius University in Bratislava, 03659 Martin, Slovakia; 4 Clinic of Neurology, University Hospital Martin, Jessenius Faculty of Medicine in Martin, Comenius University in Bratislava, 03659 Martin, Slovakia; stefan.sivak@uniba.sk (Š.S.); egon.kurca@uniba.sk (E.K.); 5 Medical Faculty, Comenius University, 81372 Bratislava, Slovakia; harsany.jan@fntt.sk; 6 Faculty of Health Care and Social Work, Trnava University, 91843 Trnava, Slovakia; denisa.jakubcova@truni.sk; 7 Department of Radiology, Faculty Hospital Trnava, 91701 Trnava, Slovakia; klepanec.andrej@fntt.sk

**Keywords:** basilar artery ischemia, aspiration thrombectomy, treatment outcome

## Abstract

**Background**: Basilar artery occlusion (BAO) is a serious disease with a poor prognosis if left untreated. Endovascular therapy (EVT) is the most effective treatment that is able to reduce mortality and disability. Treatment results are influenced by a wide range of factors that have not been clearly identified. In the present study, direct aspiration was chosen as a first-line treatment. The safety and effectiveness of direct aspiration in BAO were determined, and factors affecting patient outcomes were identified. **Methodology**: Data for patients with BAO treated between November 2013 and December 2021 were evaluated using a database. The association between clinical and procedural parameters and functional outcome was assessed. **Results**: A total of 89 patients with BAO were identified. Full recanalization was achieved in 69.7% of cases and partial recanalization in 19.1%. Intracranial hemorrhage was detected in 11 (12.4%) patients, of which, eight (9.0%) patients experienced symptomatic intracranial hemorrhage. Patients with good outcomes presented with milder strokes (mean NIHSS score of 12.58 vs. 24.00, *p* < 0.001), had higher collateral scores (6.79 vs. 5.88, *p* = 0.016), more often achieved complete recanalization (87.9% vs. 58.9%, *p* = 0.009), and more often experienced early neurological improvement (66.7% vs. 26.8%, *p* < 0.001). On the contrary, patients with worse outcomes had higher serum glucose levels (*p* = 0.05), occlusion of the middle portion of the basilar artery (MAB) (30.3% vs. 53.6%, *p* = 0.033), longer thrombus lengths (10.51 vs. 16.48 mm, *p* = 0.046), and intracranial hemorrhage (*p* = 0.035). **Conclusions**: The present study results suggest that direct aspiration is a safe and effective treatment for patients with BAO. We identified several factors affecting the patients’ outcome.

## 1. Introduction

Basilar artery occlusion (BAO) is a devastating condition associated with high mortality. It is the cause of about 1% of ischemic stroke (IS) cases and 5% of IS cases with large artery occlusion (LVO) [1,2]. The development of pharmacological and endovascular therapy (EVT) has reduced mortality and disability, but the results of treatment are still unsatisfactory. Most patients remain disabled or die [3]. Before the introduction of EVT, intravenous thrombolysis (IVT) was the only treatment option with a demonstrated efficacy and safety in randomized clinical trials [4,5]. Currently, the time indication criteria for EVT in posterior circulation are more benevolent; as it is a vital indication, patients can be treated within 24 h after symptom onset [6,7]. A meta-analysis of patients treated for LVO in the anterior circulation found that better recanalization was achieved using direct aspiration rather than a stent retriever [8,9,10]. Therefore, the present study used direct aspiration as the method of first choice.

The aim of this work was to determine the effectiveness and safety of direct aspiration as the method of first choice for BAO in a retrospective study, to identify factors that influence the resulting functional status of patients, and to compare the results with the published literature. Statistical analysis identified baseline parameters with a negative impact on the clinical outcome to be as follows: NIHSS ≥ 10 (NIHSS10), glycemia ≥ 5.60 mmol/L (Glc5.6), thrombus length ≥ 8 mm (TL8), PC-CS ≤ 7 (PCCS7), and occlusion of the middle basilar artery segment (MAB). It was hypothesized that combinations of these parameters could have an even more negative impact on the clinical outcome. Therefore, another statistical analysis was performed to identify the most significant combinations.

## 2. Methodology

The present investigation was a monocentric, non-randomized observational retrospective clinical study evaluating the safety and efficacy of direct aspiration in patients with IS caused by BAO. All patients treated with EVT for BAO that was confirmed by DSA were included in the analysis.

Baseline data were collected between November 2013 and December 2021, including information on age, sex, risk factors (history of arterial hypertension, diabetes mellitus, atrial fibrillation, ischemic cardiac disease, peripheral arterial occlusive disease, dyslipidemia, previous stroke or TIA, and malignancy), a history of antiplatelet medication and DOAC use, pre-stroke disability, neurological symptom severity, blood pressure, and serum glucose level (Table 1).

Clinical outcomes were analyzed in relation to whether the patient was brought directly to our center (mothership model) or first to the primary stroke center and consequently to our center (drip-and-ship model). Treatment with IVT and logistic data were explored in terms of the following parameters: onset-to-door time when available, door-to-image time, door-to-groin puncture time, groin puncture-to-revascularization time, and onset-to-revascularization time when available. Furthermore, radiological data analysis determined ischemic changes on non-contrast computed tomography (CT) scans, thrombus lengths, thrombus localization (proximal, middle, and distal AB segment), and collateral status (Table 2).

Data for the method of recanalization (primarily direct aspiration, stentumbra as a rescue method, primarily stent retriever, and primarily stentumbra), the use of PTA, and the number of passes were analyzed (Table 2). Recanalization status, changes in neurological status within the first 24 h after EVT, and the occurrence of all symptomatic intracranial hemorrhages on the follow-up brain CT scan were examined (Table 3).

IS etiology was also investigated (Table 4). All of the outcome data for patients three months after the treatment were summarized (Table 5).

Patient disability was evaluated using the modified Rankin scale (mRS) [11]. Neurological status was calculated according to the National Institutes of Health Stroke Scale (NIHSS) [12]. Early neurological improvement was defined as a decrease in the NIHSS score by four or more points compared to the initial NIHSS score within the first 24 h. Early neurological worsening was defined as an increase in the NIHSS score by four or more points compared to the initial NIHSS score within the first 24 h. Ischemic changes on the initial CT scans were quantified using the Posterior Circulation—Alberta Stroke Program Early Computed Tomography Score (pc-ASPECTS) [13]. Collaterals were assessed according to the posterior circulation collateral score (PC-CS) [14]. The degree of recanalization on the resulting angiogram was evaluated using the modified Treatment in Cerebral Infarction score (TICI) [15].

The etiology of IS was established according to the Trial of ORG 10172 in Acute Stroke Treatment (TOAST) classification [16]. Right–left shunt detection using a transcranial ultrasound was performed in cases without atherosclerosis, cardioembolic IS etiology, or dissection [17]. Bleeding on a follow-up brain CT scan was evaluated as symptomatic based on the ECASS 3 criteria [18].

Patients were always examined by a neurologist experienced in stroke care. Blood samples were taken for the analysis of biochemical parameters (glycemia, urea, creatinine, ionogram, osmolality, and CRP), blood count evaluation, and coagulation tests.

Imaging studies included non-contrast brain CT scans and CT angiography from the aortic arch to the vertex. Perfusion brain CT was performed in cases of unknown symptom onset. Patients were examined using a GE Revolution© CT machine. They were administered Scanlux 370© or Iomeron 400© contrast material for the CTA and perfusion CT examinations. CT findings were evaluated by an experienced radiologist.

IVT was administered at a dose of 0.90 mg/kg (10% bolus intravenously, then the remaining 90% in a continuous infusion over the course of 60 min).

Patients were then transferred to the angiosuite of the interventional radiology department. EVT was performed by experienced interventional radiologists. All procedures were performed on an angiographic device GE Innova 3100 Cath/Angio system©.

The dominant vertebral artery was probed using diagnostic catheters, followed by the introduction of a supporting sheath (Penumbra Neuron MAX©). A diagnostic DSA was performed to confirm the occlusion. Using a microcatheter (Velocity©) with a microwire (Synchro©, Portal©, Hybrid©), the closure was probed via aspiration thrombectomy (aspiration catheter 4MAX©/JETD©/ACE60©/ACE64©) as the first method of choice (ADAPT technique). If successful recanalization was not achieved after 2–3 attempts, stentumbra thrombectomy using a stent retriever (Preset©, Trevo©, Solitaire©) was performed as a “rescue”. Subsequently, a diagnostic DSA was performed in order to evaluate the effect of the treatment. In a case of unsuccessful recanalization, the entire procedure was repeated a maximum of six times.

After the procedure, all patients were transferred to the stroke unit. A non–contrast CT scan was performed 8–24 h after the procedure. 

For the purpose of data processing, the patients were divided into two groups according to the outcome, assessed using mRS scores after three months, as follows: good (mRS scores of 0–3) and bad (mRS scores of 4–6). The above parameters were compared between the two groups.

The study was approved by the Ethical Committee of Faculty Hospital, Trnava.

### Statistical Analysis

The Chi-square test was used to determine the existing differences in the frequencies of qualitative variables. Yates correction was used in the cases of frequencies lower than 10. The normality of the data distribution of quantitative variables was assessed using the Kolmogorov–Smirnov test. Since the quantitative variables were from a non-normal distribution, the mean values were compared using a two-sample independent Wilcox test.

The influence of predictors on the clinical outcome of patients was evaluated using logistic regression analysis, where mRS scores of 0–3 served as “0” and mRS scores of 4–6 as “1”. Predictors without statistical significance were excluded from the basic model using stepwise backward selection. A final multivariate model with risk and protective factors for clinically worse outcomes of the monitored patients was also compiled.

Statistical significance was determined by *p* ≤ 0.05. All data analysis was performed using SPSS statistical software, version 28.0.0.0.

## 3. Results

### 3.1. Baseline Data

A total of 1,043 patients were treated using mechanical thrombectomy between November 2013 and December 2021 at our center. Among them, there were 89 patients with BAO. There were 34 thrombectomies performed in women and 55 in men. The median age was 71 years (37–92). The median baseline NIHSS score was 19 points (3–40). Patients with a higher NIHSS score at admission had a significantly higher risk of a poor outcome three months after the procedure (*p* < 0.001). Multivariate regression analysis showed that the risk of a worse outcome increased 1.17 times with an increase in blood glucose level by 1 mmol/L. A statistical analysis of the input characteristics is shown in Table 1, while the multivariate regression analysis is in Table 6.

The median mRS score at discharge was five points. There were 33 (37.1%) patients in good clinical condition with mRS scores of 0–3 after 90 days, 24 (27.0%) patients with mRS scores of 0–2, 20 (22.5%) patients in excellent clinical condition (mRS scores of 0–1), and 14 (15.7%) patients without residual clinical symptoms (mRS scores of 0). A total of 42 (47.2%) patients died within three months. The patients’ clinical status at 90 days is summarized in Table 5.

### 3.2. Logistical Data

A total of 47 (52.8%) patients were brought directly to our center, and 42 (47.2%) were referred from primary stroke centers. The time of stroke onset was known in 55 (61.8%) patients and unknown in 34 (38.2%) patients. In the group with a known onset time, the average time of symptom onset after arrival (OTD time) to the treatment center was 277 min (30–1,630). By comparing patients with good and poor clinical status, it was found that the OTD time did not significantly affect patient outcome (255 min for an mRS score of 0–3 vs. 292 min for an mRS score of 4–6, *p* = 0.479).

### 3.3. Radiological Parameters

Statistically significant factors, such as the length of the thrombus and collateral score, were identified. Patients with a longer thrombus (16.48 mm vs. 10.51 mm, *p* = 0.057) were more often associated with a worse outcome. Regression analysis results showed that if the thrombus length increased by 1 mm, the chance of a worse outcome increased 1.06 times. Patients who had a lower collateral score (5.88 vs. 6.79, *p* = 0.016) had a significantly worse outcome. Regression analysis demonstrated that if the value of the collateral score increased by one point, the chance for a worse outcome decreased 0.68 times. The differences in pc-ASPECT values were not significant (9.09 vs. 9.09, *p* = 1). An occlusion location in the MAB was significantly more frequent in patients with a worse resulting clinical condition, and regression analysis showed that patients with a closed MAB had a higher risk of a worse outcome (*p* = 0.033). The chance of a better outcome was 0.68 times lower in cases of MAB occlusion.

IVT was administered to 38 patients (42.7%). In the group with mRS scores of 0–3, 14 (42.4%) patients were treated with IVT. A total of 24 (42.9%) patients were treated in the group with mRS scores of 4–6, which was not significantly different (*p* = 1).

Direct aspiration (ADAPT technique) was the primary method used in the present study. The stentumbra technique was used as a rescue when direct aspiration failed. The aspiration catheter could not be inserted in 11 (12.4%) cases due to unfavorable anatomy. The stentumbra technique was used directly in one (1.1%) case. A stent retriever was the primary choice in seven (7.9%) cases. The artery was recanalized in two (2.2%) cases after using balloon angioplasty alone. Brachial artery access had to be used in 10 (11.2%) patients. The average time from groin puncture to recanalization was 23.3 min. PTA was used in 18 (20.2%) patients and a stent was implanted in six (9.2%) patients. The logistic data and radiological findings are summarized in Table 2.

### 3.4. Safety and Efficiency Data

TICI 3 recanalization was achieved in 62 (69.7%) cases and TICI 2b in 17 (19.1%) cases. TICI 3 recanalization using direct aspiration was achieved in 52 (58.4%) cases and TICI 2b in 13 (14.6%) cases. The stentumbra technique had to be used as a rescue method to achieve successful recanalization in 11 (12.4%) cases. Recanalization at level 2a was achieved in one (1.1%) case. Recanalization (TICI 0) was not achieved in nine (10.1%) cases.

The average time from groin puncture to recanalization was 23.31 min, which was not significantly different between the groups (23.20 min for mRS scores of 0–3 and 23.38 min for mRS scores of 4–6, *p* = 0.343). In the case of known time of symptom onset, the appropriate time from onset to recanalization was 338.45 min, and there was no significant difference between patients with good and poor outcomes (324.43 min for mRS scores of 0–3 and 349.96 min for mRS scores of 4–6, *p* = 0.705).

Within 24 h after the procedure, 37 (41.6%) patients improved by four or more NIHSS points. On the contrary, 20 (22.5%) patients worsened by four or more NIHSS points. The resulting clinical condition of patients who improved by four or more points was significantly better compared to those who did not (66.7% vs. 26.8%, *p* < 0.001). Patients who improved by more than four points were 5.47 times more likely to have a good clinical status than patients who improved by three or fewer NIHSS points, and their clinical status did not change or deteriorated.

Intracranial hemorrhage was detected in 11 (12.4%) patients on the control CT scans, of which eight (9.0%) patients experienced symptomatic intracranial hemorrhage. No patient with a good clinical outcome had a symptomatic intracranial hemorrhage. Regression analysis showed that intracranial bleeding on the follow-up CT examination was associated with a 10-fold reduction in a chance of a good clinical outcome. Mortality 3 months (mRS 6) after treatment was 47.2%. Data on the efficacy and safety of endovascular treatment are shown in Table 3 and Table 6.

### 3.5. Etiology of Stroke

Cardioembolization was the most common cause of IS in 28 (31.5%) cases, followed by atherothrombotic etiology in 25 patients (28.1%), dissection in five (5.6%) patients, and paradoxical embolization through the foramen ovale aperture in two (2.2%) cases. The cause could not be determined during the monitored period in 29 (32.6%) patients. The etiology data are summarized in Table 4.

## 4. Discussion

Several clinical studies and their meta-analyses have confirmed the effectiveness and safety of EVT in patients with occlusion of large arteries in the anterior circulation [5]. A much smaller number of clinical studies have investigated EVT in the posterior circulation [19] Documented clinical studies of the posterior circulation have shown that patients with vertebrobasilar IS have a lower chance of recanalization with a higher risk of complications and mortality, as well as a worse clinical outcome, when compared to those undergoing anterior circulation treatment [20]. These results have been confirmed by a meta-analysis of 21 studies comparing the effects of recanalization treatment between the anterior and posterior circulations [21].

A statistically significant benefit of EVT in patients with acute BAO has also been confirmed in a meta-analysis of 102 clinical observational studies published by Sheng and Tong. Data showed that EVT is safer and more effective than intra-arterial thrombolysis (IAT) and IVT. However, this meta-analysis had a number of limitations, as it was an observational clinical study with a heterogeneous design and a small number of patients. 

To date, four randomized prospective multicenter open-label clinical trials have been published comparing the efficacy of EVT + BMT vs. BMT alone. The BEST clinical trial did not demonstrate a statistically significant difference in the number of patients with mRS scores of 0–3 after 90 days in the group of patients treated using mechanical thrombectomy (42% and 32%, respectively). Both groups had the same mortality (33.3% vs. 38.5%), and the incidence of symptomatic intracranial bleeding was higher in the EVT group (0 vs. 8%) [22].

The BASICS study also did not show a significant difference between EVT procedures, and there were no significant differences in mortality and symptomatic intracranial bleeding [23].

In contrast to previous studies, the ATTENTION clinical trial demonstrated that EVT was significantly more effective compared to BMT alone (mRS scores of 0–3 present in 46.0% vs. 22.8% of the study population, respectively). The 90-day mortality was significantly lower in the EVT arm. Intracranial hemorrhage occurred significantly more often in the group with EVT (5.3% vs. 0%) [7]. BAOCHE was the second positive randomized multicenter clinical trial. After 90 days, 46.4% of patients treated with EVL had an mRS score of 0–3, in contrast to 24.3% of the BMT patients. The difference in mortality was in favor of EVT (30.9% vs. 42.1%). There was also an insignificant difference in symptomatic hemorrhage in favor of BMT (5.9% vs. 2.1%) [6].

The benefit of EVT was confirmed by the results of a prospective non-randomized multicenter registry, BASILAR. In the group treated with EVT, significantly more patients achieved mRS scores of 0–3 after 90 days (32% vs. 9.3%), and mortality was significantly lower in the group treated with EVT (46.2% vs. 71.4%), despite a significantly higher incidence of symptomatic intracranial bleeding (7.1% vs. 0.5%) [24].

Factors associated with recanalization and the resulting clinical status of patients with acute BAO undergoing endovascular treatment using a stent retriever were evaluated in the prospective registration study, ENDOSTROKE. TICI 2b and 3 recanalization was achieved in 79% of patients [25]. In a retrospective by van Houwelingen et al., recanalization was achieved in 89.5% of patients using stent retrievers [26]. Kang et al. have achieved the recanalization of TICI 2b and 3 in 91.5% of patients in a multicenter retrospective observational study. A stent retriever was primarily used in 68.4% of patients [27]. Another monocentric retrospective analysis by Baek et al. had a similar EVT design, where TICI level 2b and 3 recanalization was achieved using the Solitaire stent retriever in 84% of patients [28]. Two small retrospective analyses comparing stent retrievers to contact aspiration found that complete recanalization was more often and more quickly achieved with direct aspiration [29,30].

In the present study, 37.1% of patients achieved a good clinical status after 90 days, with mRS scores of 0–3. In randomized trials, a good clinical status was achieved in 46% of patients in the ATTENTION study, 46% in the BAOCHE study, 44% in the BEST study, and 44.2% in the BASICS study. Registry results were 42% for the ENDOSTROKE and 32% for the BASILAR. The rate of TICI 2b and 3 recanalization in patients treated with EVT was 72% to 88% in these studies, respectively. In the present study group of patients, recanalization was achieved in 73% of individuals using only the ADAPT technique. TICI 2b and 3 recanalization using rescue stentumbra was achieved in 88.8% of cases, which agrees with the published data. The incidence of symptomatic intracranial hemorrhage was 9% in the present study group and 4.5–7.1% in the above studies. Our results (mRS scores after 90 days, degree of recanalization, and occurrence of intracranial bleeding) are comparable with the results of the randomized prospective clinical trials ATTENTION, BAOCHE, BEST, and BASICS and the registration studies BASILAR and ENDOSTROKE. These results are expected to be closest to the results of the BASILAR prospective registration study due to the similar patient selection process, which is comparable to the real-world practice. In contrast to the prospective registry, the present study’s EVT center also treated patients with a more severe disability before IS, i.e., patients with pre-stroke mRS scores of more than two, as well as patients with advanced oncological conditions [6,7,22,23,24,25].

In most published works, patients with a lower initial NIHSS score had a better outcome than patients with a higher value [31,32,33]. The present study patients had higher initial NIHSS scores compared to patients in other published works, which may explain the higher mortality in the present cohort.

Hyperglycemia is associated with a worse outcome for patients with IS. It is an independent predictor of a larger stroke volume and increases the risk of death. This was also demonstrated by meta-analysis in patients treated with mechanical thrombectomy and was also valid for patients with BAO in the present study group [34].

Kwak and Park have published a study in which patients more often had better outcomes with distal or proximal AB occlusion [9]. On the contrary, other authors did not show any significant association between AB occlusion location and patient outcome [24,27,32,35]. In the present study, patients had a worse outcome if they had a MAB lesion.

Kong et al. have not found a significant connection between the value of PC-CS and the resulting patient state. On the contrary, patients in the study by Kwak and Park had a worse outcome with a decreasing collateral score, which was similar to the present study results [9,35].

Two prior studies have explored the influence of the thrombus length on the patient’s outcome. Neither Shu et al. nor van der Hoeven et al. have found a significant association between the thrombus length and the resulting clinical status [36,37]. These outcomes are in contradiction with the present study results, which demonstrated the dependence of the final patient status on the thrombus length.

Occluded artery recanalization is the basic premise of achieving a good clinical outcome in patients after IS, patients have a poor outcome if the vessel does not pass [5,9,32,33,35,38]. The present results also confirmed this hypothesis and a statistically significant difference was achieved with complete recanalization (TICI 3).

Early neurological improvement was shown to be a strong independent predictor of good outcome both in patients treated with EVT for LVO and in patients with BAO, which was also confirmed by Wang et al., Guenego et al., Alexandre et al., and the present study’s authors [39,40,41].

Symptomatic intracranial hemorrhage as a complication of EVT, and specifically in BAO, significantly worsens the resulting clinical condition of patients treated with EVT. A statistically significant negative impact of any intracranial hemorrhage was demonstrated in only a few prior works [35,38].

The statistical analysis of risk parameter combinations was performed based on the univariate analysis results. Several combinations significantly worsened the outcome. Of the observed parameters, MAB occlusion appeared to be the worst predictor, as it was present in most of the combinations.

The present study had several limitations. First, this was a retrospective study, and the data were obtained from a relatively small number of patients from one center. Furthermore, a control group of patients was missing. Finally, a rescue treatment was used, which could have affected the treatment results.

To the best of our knowledge, this is the largest published data set of patients with acute BAO treated with primary aspiration thrombectomy in Slovakia that was used to identify several outcome predictors. Given that Slovakia has a relatively high incidence of stroke, efforts should be targeted not only at primary prevention, but also at other potentially influential factors in order to improve patient outcomes.

## 5. Conclusions

Randomized clinical trials have only recently confirmed the statistically significant effectiveness of EVT and the resulting clinical condition. The present monocentric retrospective analysis confirmed the safety of direct aspiration in the treatment of acute BAO and its effectiveness in achieving revascularization. Based on these results, we support the use of direct aspiration as the first-line treatment for BAO when possible.

Statistical analyses demonstrated that a lower baseline NIHSS score, higher collateral score, complete recanalization, and an early neurological improvement are predictors of a good outcome. On the contrary, a higher glycemia level, MAB occlusion, longer thrombus length, and the presence of bleeding on a follow-up brain CT scan are predictors of a poor clinical outcome. Several combinations of risk parameters that negatively affected patient outcomes and were most aggravated by MAB occlusion were also identified. Worse treatment results can be expected in the presence of these parameters or their combinations, although that should not serve as a reason not to provide EVT.

Further randomized controlled trials are needed to evaluate the effectiveness and safety of direct aspiration and to compare it to stent retriever treatment, in order to determine the best strategy for performing mechanical thrombectomy. In addition, larger clinical trials are needed to confirm the data on prognostic factors and their combinations.

## Figures and Tables

**Table 1:** Baseline data.

	Total	mRS 0–3	mRS 4–6	*p*-Value
*n*, (%)	89 (100.0%)	33 (37.1%)	56 (62.9%)	-------
Age (average ± SD; median)	69.91 ± 11.39; 71.00	69.12 ± 10.98; 71.00	70.38 ± 11.70; 71.50	0.619
Men, *n* (%)	55 (61.8%)	17 (51.5%)	38 (67.9%)	0.191
Women, *n* (%)	34 (38.2%)	16 (48.5%)	18 (32.1%)	
Arterial hypertension, *n* (%)	78 (87.6%)	28 (84.8%)	50 (89.3%)	0.779
Diabetes mellitus, *n* (%)	17 (19.1%)	4 (12.1%)	13 (23.2%)	0.314
Atrial fibrillation, *n* (%)	28 (31.5%)	7 (21.2%)	21 (37.5%)	0.173
Ischemic heart disease, *n* (%)	28 (31.5%)	7 (21.2%)	21 (37.5%)	0.173
Peripheral artery disease, *n* (%)	8 (9.0%)	2 (6.1%)	6 (10.7%)	0.721
Hyperlipidemia, *n* (%)	45 (50.6%)	21 (63.6%)	24 (42.9%)	0.094
Previous stroke, TIA, *n* (%)	18 (20.2%)	4 (12.1%)	14 (25.0%)	0.235
Malignity, *n* (%)	5 (5.6%)	3 (9.1%)	2 (3.6%)	0.538
Antiplatelet treatment	33 (37.1%)	11 (33.3%)	22 (39.3%)	0.954
Direct oral anticoagulation treatment	9 (10.1%)	1 (3.0%)	8 (14.3%)	0.181
Pre-stroke mRS (average ± SD; median)	0.29 ± 0.92; 0.00	0.21 ± 0.86; 0.00	0.34 ± 0.96; 0.00	0.2745
Baseline NIHSS (average ± SD; median)	19.76 ± 11.80; 19.00	12.58 ± 9.59; 9.00	24.00 ± 10.95; 26.00	0.001
Systolic BP (average ± SD; median)	158.44 ± 28.22; 160.00	164.00 ± 26.55; 160.00	154.70 ± 29.00; 160.00	0.272
Diastolic BP (average ± SD; median)	84.19 ± 15.36; 80.50	85.55 ± 14.90; 80.00	83.28 ± 15.77; 82.00	0.926
Glycemia (average ± SD; median)	8.80 ± 4.13; 7.70	7.55 ± 2.03; 7.49	9.52 ± 4.82; 8.14	0.189

**Table 2:** Radiological and logistical data.

	Total	mRS 0–3	mRS 4–6	*p*-Value
*n*, (%)	89 (100%)	33 (37.1%)	56 (62.9%)	-------
Mothership model, *n* (%)	47 (52.8%)	15 (45.5%)	32 (57.1%)	0.286
Drip-and-ship model, *n* (%)	42 (47.2%)	18 (54.5%)	24 (42.9%)	0.286
Onset-to-door (average ± SD; median)	276.95 ± 259.26; 220	255.35 ± 217.49; 205	292.47 ± 287.96; 245	0.479
Door-to-imaging (average ± SD; median)	15.82 ± 17.44; 10	15.07 ± 14.45; 10	16.28 ± 19.20; 10	0.885
Door-to-groin puncture (average ± SD; median)	154.78 ± 100.84; 133	147.19 ± 76.35; 145	159.90± 115.13; 129	0.238
Groin-to-revascularization (average ± SD; median)	23.31 ± 14.07; 19.50	23.20 ± 16.66; 17	23.38 ± 12.11; 21	0.343
Onset-to-revascularization (average ± SD; median)	338.45 ± 269.68; 284	324.43 ± 235.66; 275	349.96 ± 298.53; 289	0.705
Pc-ASPECTS (average ± SD; median)	9.09 ± 1.28; 10	9.09 ± 1.26; 10	9.09 ± 1.31; 10	1.000
Thrombus length (average ± SD; median)	14.27 ± 11.94; 10	10.51 ± 7.29; 9.50	16.48 ± 13.56; 12.75	0.046
Proximal basilar artery occlusion, *n* (%)	30 (33.7%)	8 (24.2%)	22 (39.3%)	0.223
Middle basilar artery occlusion, *n* (%)	40 (44.9%)	10 (30.3%)	30 (53.6%)	0.033
Distal basilar artery occlusion, *n* (%)	59 (66.3%)	23 (69.7%)	36 (64.3%)	0.772
PC-CS (priemer ± SD; median)	6.21 ± 1.61; 6	6.79 ± 1.54; 7	5.88 ± 1.57; 6	0.016
IVT, *n* (%)	38 (42.7%)	14 (42.4%)	24 (42.9%)	1.000
Direct aspiration, *n* (%)	77 (86.5%)	31 (93.9%)	46 (82.1%)	0.116
Rescue stentumbra, *n* (%)	12 (13.5%)	6 (18.2%)	6 (10.7%)	0.642
Stentretriever, *n* (%)	7 (7.9%)	2 (6.1%)	5 (8.9%)	0.819
Primary stentumbra, *n* (%)	1 (1.1%)	1 (3.0%)	0 (0.0)	NA
PTA, *n* (%)	18 (20.2%)	6 (18.2%)	12 (21.4%)	0.790
Number of passes (average ± SD; median)	1.71 ± 1.14; 1	1.48 ± 0.85; 1	1.85 ± 1.29; 1	0.238

Pc-ASPECTS (Posterior Circulation—Alberta Stroke Program Early Computed Tomography Score), PC-CS (posterior circulation collateral score), intravenous thrombolysis (IVT).

**Table 3:** Safety and efficiency data.

	Total	mRS 0–3	mRS 4–6	*p*-Value
*n*, (%)	89 (100.0%)	33 (37.1%)	56 (62.9%)	-------
TICI 2b and 3, *n* (%)	79 (88.8%)	32 (97.0%)	47 (83.9%)	0.125
TICI 3, *n* (%)	62 (69.7%)	29 (87.9%)	33 (58.9%)	0.009
TICI 2b, *n* (%)	17 (19.1%)	3 (9.1%)	14 (25.0%)	0.118
TICI 2a, *n* (%)	1 (1.1%)	0 (0.0%)	1 (1.8%)	NA
TICI 1, *n* (%)	0 (0.0%)	0 (0.0%)	0 (0.0%)	NA
TICI 0, *n* (%)	9 (10.1%)	1 (3.0%)	8 (14.3%)	0.181
Early neurological improvement, *n* (%)	37 (41.6%)	22 (66.7%)	15 (26.8%)	0.001
Early neurological deterioration, *n* (%)	20 (22.5%)	0 (0.0%)	20 (35.7%)	NA
Any intracranial bleeding, *n* (%)	11 (12.4%)	1 (3.0%)	10 (17.9%)	0.086
Symptomatic intracranial bleeding, *n* (%)	8 (9.0%)	0 (0.0)	8 (14.3%)	NA

Modified Treatment in Cerebral Infarction score (TICI).

**Table 4:** Ischemic stroke etiology.

	Total	mRS 0–3	mRS 4–6	*p*-Value
*n*, (%)	89 (100.0%)	33 (37.1%)	56 (62.9%)	-------
Atherothrombotic, *n* (%)	25 (28.1%)	9 (27.3%)	16 (28.6%)	0.895
Cardioembolic, *n* (%)	28 (31.5%)	9 (27.3%)	19 (33.9%)	0.514
Dissection (*n*, %)	5 (5.6%)	3 (9.1%)	2 (3.6%)	0.280
Patent foramen ovale (*n*, %)	2 (2.2%)	1 (3.0%)	1 (1.8%)	0.692
Undetermined (*n*, %)	29 (32.6%)	11 (33.3%)	18 (32.1%)	0.908

**Table 5:** Clinical outcome.

	*n*, %
mRS 0	14, 15.7%
mRS 1	6, 6.7%
mRS 2	4, 4.5%
mRS 3	9, 10.1%
mRS 4	6, 6.7%
mRS 5	8, 9.0%
mRS 6	42, 47.2%
mRS 0–1	20, 22.5%
mRS 0–2	24, 27.0%
mRS 0–3	33, 37.1%
mRS 4–6	56, 62.9%

Modified Rankin scale (mRS).

**Table 6:** Risk and protective factors of clinically worse patient outcome.

Risk Factors	Coefficient	OR	95%CI	*p*-Value
Baseline NIHSS	0.10	1.11	1.05–1.16	0.001
Glycemia	0.15	1.17	1.00–1.36	0.050
Thrombus length	0.06	1.06	1.01–1.12	0.026
Early neurological improvement—yes	1			
Early neurological improvement—no	1.70	5.47	2.14–13.92	0.001
**Protective Factors**	**Coefficient**	**OR**	**95%CI**	***p*-Value**
Collateral status	−0.39	0.68	0.50–0.92	0.012
Middle basilar artery occlusion—yes	1			
Middle basilar artery occlusion—no	−0.97	0.38	0.15–0.94	0.035
Any intracranial bleeding—yes	1			
Any intracranial bleeding—no	−2.28	0.10	0.01–0.85	0.035

National Institutes of Health Stroke Scale (NIHSS).

## Data Availability

Data are contained within the article.
